# How Cultural Behaviors and Superstitions Associate the Willingness to Undergo Cataract Surgery in Taiwan: A Nationwide Survey

**DOI:** 10.3390/medicina59050973

**Published:** 2023-05-18

**Authors:** Hsiao-Fan Tung, Yi-Ling Chen, Chiu-Liang Chen, Mei-Jih Gee, Chih-Hsin Muo, Shin-Lin Chiu

**Affiliations:** 1 Department of Ophthalmology, Changhua Christian Hospital, Changhua 500, Taiwan; 2 Surgery Clinical Research Center, Changhua Christian Hospital, Changhua 500, Taiwan; 3 Department of Orthopedics, Changhua Christian Hospital, Changhua 500, Taiwan; 4 Department of Nursing, Hungkuang University, Taichung 433, Taiwan; 5 Department of Statistics, Feng-Chia University, Taichung 407, Taiwan; 6 Management Office for Health Data, China Medical University Hospital, Taichung 404, Taiwan; 7 College of Nursing and Health Sciences, Da-Yeh University, Changhua 515, Taiwan; 8 Department of Post-Baccalaureate Medicine, College of Medicine, National Chung Hsing University, Taichung 402, Taiwan

**Keywords:** cultural behaviors, superstitions, medical decision making, ghost month belief, cataract surgery, gender disparity

## Abstract

*Background and Objectives*: Cultural beliefs influence the conceptualization, adaptation, and coping strategies for diseases. This study aimed to investigate the impact of cultural beliefs and customs on the willingness to undergo cataract surgery in Taiwan. *Materials and Methods*: The data were retrospectively retrieved from the national Longitudinal Health Insurance Database 2000 (LHID2000). From the national database, we enrolled patients that were diagnosed with cataracts and underwent cataract surgery from 2001 to 2010. All the patients were stratified according to their gender and living area. Gender was categorized as male or female, and the living area was classified as urban or rural. We compared the difference in the number of surgeries between stratified patient groups in each Chinese lunar month. *Results*: The number of cataract surgeries decreased significantly in the seventh and twelfth lunar months for both genders. There was a significant reduction in cataract surgeries in both the urban and rural groups during the seventh lunar month. Interestingly, only the seventh lunar month had an association with sex in different living areas, which meant that in the seventh month, there was a gender-specific difference in the surgical numbers. *Conclusions*: The Taiwanese population holds a belief that surgical procedures, including cataract surgery, during the lunar ghost month is inauspicious. Citizens tend to avoid elective surgery due to cultural practice, resulting in a decrease in surgical numbers during the period of the Chinese New Year. The authorities should consider these cultural behaviors when developing medical policies and allocating resources. Healthcare providers should be aware of these superstitions and take them into account when providing medical care and advice to patients.

## 1. Introduction

The impact of cultural beliefs on healthcare-seeking behavior has been documented in various studies all around the world. For instance, studies have shown that cultural beliefs and attitudes toward illness affect the decision to seek healthcare, the type of treatment preferred, and the level of trust in healthcare providers [1,2,3,4,5,6]. Moreover, cultural beliefs and practices can also influence the adherence to treatment regimens and the perception of the effectiveness of medical interventions. On the other hand, understanding the impact of cultural beliefs on healthcare-seeking behavior is important for healthcare providers as it can help them provide culturally sensitive care and improve patient reliability and outcomes. Medical providers who are aware of cultural beliefs and practices can tailor their communication and treatment plans to better align with the patient’s values and preferences. This can improve the satisfaction of patients, result in higher adherence to treatment and improve overall health outcomes.

The Chinese lunar calendar and festivals have a significant influence on Asian cultures, not just in China, but also in other countries with sizable Chinese populations such as Taiwan, Hong Kong, Singapore, and Malaysia. The Chinese lunar year begins with the first new moon of the lunar calendar, usually in late January or early February. Each lunar month is associated with various cultures, festivals, and traditional beliefs, which are deeply rooted in Chinese culture. Among all the Chinese festivals, in Taiwan, Chinese New Year, the Qingming Festival (also called Tomb Sweeping Day), Moon Festival, and Mid-Autumn Festival are the most important ones. Due to traditional customs, national holidays are usually observed during these festivals, with five days for Chinese New Year and one day for the others. The period from the end of the twelfth lunar month to the next new lunar year is the most significant festival in Chinese culture. This time, also known as “Chinese New Year” or the “Spring Festival,” is a time of family gathering, celebration, and joy. The celebrations last for days and include various traditional customs and activities, such as worshiping the ancestors, giving red envelopes with money in to children, and preparing and enjoying traditional foods. People used to be blessed but busy in this period, due to lots of kinds of customs activities.

In contrast, the seventh lunar month is known as the “Ghost Month”, when it is believed that the spirits of the dead return to the world of the living. During this month, there is no specific national holiday. Many Chinese people avoid making important decisions, getting married, or undergoing surgery because of the belief that it is an inauspicious time. It is similar to “Friday the 13th” in the United States.

In this study, we examined the relationship between cultural beliefs and medical decision-making by analyzing the association of the Chinese lunar months on the number of cataract surgeries performed in Taiwan. Cataract surgery was chosen as the index of procedure as it is typically an elective procedure that is not affected by seasonal or weather-related factors. In Taiwan, cataract surgery is covered by the national health insurance by the government and there is no difference in the offers from health facilities each month. In addition, the decision to undergo cataract surgery is typically based on the degree of visual impairment and its impact on the patient’s quality of life. Modern cataract surgery is performed as an outpatient under local anesthesia and used to take less than half an hour. Cataract operations make up most ophthalmic surgery cases and are readily available in Taiwan [7,8,9,10]. As a result, we believe that the number of cataract surgeries performed in Taiwan is a good indicator of cultural-related changes in medical-seeking behavior. To analyze the relationship between the Chinese lunar months and the patients’ willingness to undergo cataract surgery, we conducted a study using nationwide data to analyze the relationships between different Chinese lunar months and the number of cataract surgeries that took place.

## 2. Materials and Methods

This is a retrospective study, designed as a population-based review using the national Longitudinal Health Insurance Database 2000 (LHID2000). The LHID2000 is a valuable resource for conducting population-based studies in Taiwan, as it provides a representative sample of the population and allows for the investigation of various health conditions and interventions. The data contained in LHID2000 are randomly selected from a million subjects from Taiwan’s National Health Insurance Research Database (NHIRD) and made available for research purposes. Both the LHID2000 and NHIRD contain a lot of computerized records, including registration files, medication and treatment regimens, and information on surgery, which are provided to researchers in an electronically encrypted form. We officially obtained approval for the data obtained from the LHID2000. From the database, we collected the patients’ identification numbers, gender, diagnostic codes, operative procedure codes, dates for the operative procedure, and living areas. The diagnostic codes for the diseases are defined by the International Classification of Diseases, 9th Revision, Clinical Modification (ICD-9-CM366) and the surgical procedure codes are defined according to the International Classification of Diseases operative procedure code (ICD-OP). The study was approved by the Institutional Review Board of Changhua Christian Hospital and all the research was performed in accordance with the relevant guidelines and regulations. This study was conducted in accordance with the Declaration of Helsinki and other relevant ethical guidelines.

We enrolled patients with a diagnosis of cataracts, who underwent cataract surgery from 2001 to 2010. The number of cataract surgeries was counted according to the lunar months. To investigate whether gender and living area had any association on the frequency of cataract surgeries during different lunar months, the patients were stratified based on these two variables. Gender was categorized as male or female, and living area was classified as urban or rural. This allowed for more in-depth analysis of the potential factors influencing the frequency of cataract surgery during different lunar months.

The statistical analysis was performed using SPSS software, version 23.5. We used the Wilcoxon signed rank test to compare the number of surgeries in each month vertically in all patients and each stratified group. The Mann–Whitney U tests and the Kruskal–Wallis test were applied to analyze differences in the frequency of cataract surgeries in stratified groups during different lunar months. Furthermore, we also utilized heterogeneous AR (1), a linear mixed model with a covariance structure, for the longitudinal data to investigate the differences in the frequency of cataract surgeries in the stratified groups during different lunar months. As the numbers of surgeries in conjoint lunar months were correlated, and the variance in each lunar month may be different, the covariance was set as heterogeneous AR (1) in the linear mixed model. The F statistic was used to test whether two or more means were equal. A *p* value of <0.05 was considered to indicate statistical significance, and all tests were two-tailed.

## 3. Results

**Figure 1:**
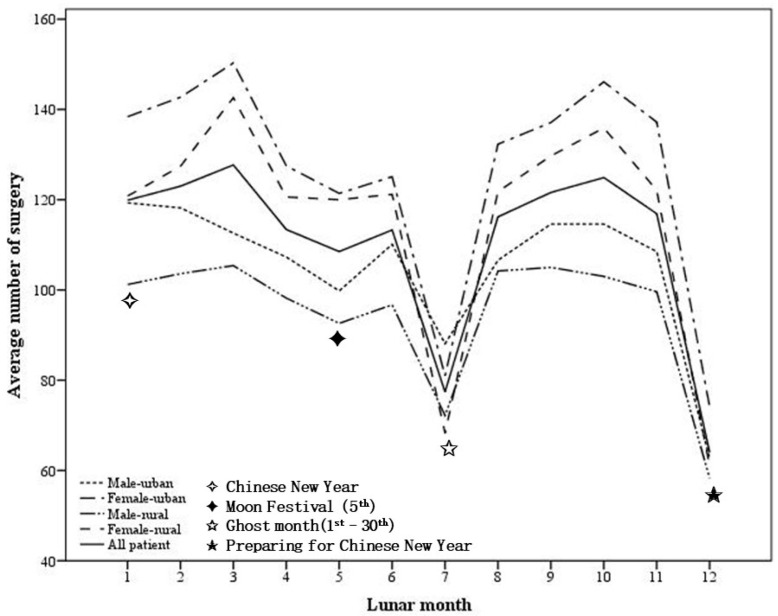
The average number of cataract surgeries in the stratified groups during different Chinese lunar months. There were drops in the seventh and twelfth Chinese lunar months in the number of cataract surgeries in all stratified groups.

The average number of cataract surgeries in different lunar months from 2001 to 2010 are illustrated in Figure 1. The mean number of cataract surgeries was 110.56 per 1,000,000 citizens per month. The number of surgeries varied across the lunar months, with the seventh and twelfth lunar months showing significantly lower numbers compared to the other months (seventh lunar month: mean = 77.40 ± 13.89, *p* < 0.001; twelfth lunar month: mean = 64.00 ± 24.50, *p* < 0.001). This finding suggests that the lunar calendar might influence the frequency of cataract surgeries in Taiwan, with fewer surgeries occurring during certain months of the year. Additionally, gender was found to be a significant factor in the frequency of cataract surgeries. Women underwent significantly more cataract surgeries than men in all lunar months, except for the seventh and twelfth months (seventh lunar month: *p* = 0.193, twelfth lunar month: *p* = 0.323).

When analyzing the association of the living environment on cataract surgery, only in the seventh lunar month was there a significant difference between urban and rural areas. In the seventh lunar month, the number of cataract surgeries in the rural area was much lower than in the urban areas (*p* < 0.001) (Table 1). We also conducted separate analyses for males and females and found that in both groups, the number of cataract surgeries significantly decreased during the 7th lunar month compared to all other months (*p* < 0.01), with the exception of the 12th month in females (in the 12th month, *p* = 0.513).

In the mixed model analysis, we chose the 1st, 7th, and 12th lunar months for comparison with the other lunar months due to the 1st and 12th lunar months representing the Chinese New Year and the 7th representing ghost month. From the results in Table 2, the interaction of gender with the seventh lunar month was significant (urban *p* < 0.001, rural *p* < 0.001); however, the interaction of gender with the twelfth lunar month was not significant (urban *p* = 0.146, rural *p* = 0.064). The above finding revealed that in the seventh month, there was a gender-specific difference in the surgical numbers.

## 4. Discussion

Our study highlights the significant association of cultural behavior on medical decisions, even for relatively safe and quick outpatient surgeries, such as cataract surgery. We found a significant reduction in the number of cataract surgeries during the Chinese seventh and twelfth months. It is worth noting that the population in Taiwan consists of more females than males, with a mean sex ratio of 96.51 from 2001 to 2010, according to official data from the Department of Household Registration in Taiwan. Furthermore, the Ministry of Interior of Taiwan reports that women have a longer life expectancy than men. The average life expectancy for women in Taiwan increased from 78.1 years in 2001 to 80.8 years in 2010, while men’s average life expectancy increased from 72.7 years in 2001 to 76.2 years in 2010. The above demographic factors might have contributed to the higher number of cataract surgeries in women.

Cultural beliefs influence the conceptualization, adaptation, and coping strategies for diseases [1]. The influence of cultural beliefs on healthcare is a global phenomenon. In Germany, for example, more than 10% of the population believes in the relationship between diseases and the lunar phase [2]. Dr. Vance also reported that 43% of health professionals believed that individual human behavior was affected by lunar effects in 1995 [3]. In the United States, superstitions such as the belief that Friday the 13th is an unlucky day can also impact behavior. Some people may avoid making important decisions or taking risks on this day, while others may alter their daily routines, such as avoiding travel or staying indoors [4,5]. It is clear that various kinds of superstitions can influence human behavior in different ways. In 2006, Dr. Becenti announced that cultural beliefs affect medical decision making. Cultural beliefs and practices can influence how individuals perceive and respond to medical issues, ranging from preventive health behaviors to medical treatment choices [6]. This draws attention to the importance of understanding and acknowledging the cultural and social factors that shape medical practices and patient behavior. It is crucial for medical providers to recognize the impact of cultural beliefs and superstitions on patient behavior and medical decision making. By understanding the cultural factors that influence patients, healthcare professionals can provide more culturally sensitive care and improve health outcomes. It is, therefore, essential to encourage open communication between patients and healthcare providers to ensure that cultural beliefs also play an important role in medical practices.

In southern China, Hong Kong, and Taiwan, the seventh lunar month is known as the “Chinese Lunar Ghost Month” or “Hungry Ghost Festival”. During this month, it is believed that the gates of the afterlife are opened, and the spirits of the dead return to the living world. The lunar ghost month is believed to be a time of heightened spiritual activity. The cultural belief is that during the lunar ghost month, the world is filled with wandering spirits, and the spirits can cause accidents, unfortunate events, or harm to people. As a result, many people in Taiwan avoid certain activities during this time, including swimming, getting married, moving into a new house, starting a new business, and may postpone major surgical procedures until the end of this month [11,12,13]. What is more, similar to Friday the 13th in the U.S., the Taiwanese avoid unnecessary risky activities to keep them away from bad luck and protect themselves from being hurt in the seventh lunar month [12]. Concerning medical treatment, people tend to avoid hospitalization for elective surgery. Previous studies have shown that cesarean delivery rates are significantly lower during the lunar month of July and higher than average during June. This is thought to reflect preemptive cesarean deliveries that are scheduled in advance in order to avoid delivery during the lunar ghost month. Women may opt for elective cesarean deliveries in order to ensure that their delivery does not occur at a time that is considered inauspicious and to prevent their baby from being unlucky during the lunar ghost month [13,14]. On the other hand, choosing an auspicious date to deliver also plays an important role when Chinese women decide on their way of delivery [14,15]. In 2018, Dr. Chiu conducted a study comparing elective total knee replacement and emerging proximal femur fracture surgery in Taiwan. The number of elective total knee replacements decreased only in the lunar ghost months [16]. Our study revealed similar observations and trends to those in related research.

In addition to the ghost month, the number of surgeries for cataracts was also reduced in the twelfth lunar month. This might be due to another custom. The twelfth lunar month is followed by Chinese New Year. It is a time for family gatherings, feasting, and celebrating the start of a new year. People believe that the New Year will bring good luck and that they cannot be absent from this festival. Patients may also be concerned about post-surgical recovery during a time when they would prefer to be spending time with their family and participating in festive activities. As a result, the turn-of-the-year effect also makes the Taiwanese avoid elective surgery due to the festive atmosphere and desire to spend time with family. The result was also similar to the one which Dr. Chiu reported [16]. All of the above findings show that cultural beliefs play an important role and influence medical decision making, especially when it is not emergent and people can “choose the time.” In addition, in Taiwan, family reunions are a crucial cultural practice during Chinese New Year. This celebration is an important event for people to gather with their families, eat traditional foods, and participate in cultural activities. Consequently, many healthcare providers take time off to celebrate this holiday with their families. This leads to reduced staffing levels and longer waiting times for patients seeking medical care, including surgery. As a result, the number of surgeries performed during the Chinese New Year period may be influenced by this cultural practice. Patients may choose to delay their surgery until after the holiday season to avoid longer waiting times or missing traditional activities.

The present study makes an important contribution to the existing literature by, firstly, exploring the relationships between sociocultural factors and outpatient surgery using a population-based and nationwide database. The finding of this study shed light on the impact of sociocultural factors on healthcare behavior in Taiwan. The results demonstrate that cultural beliefs and taboos surrounding specific lunar months can significantly influence outpatient surgery rates, with a reduction in cataract surgeries during the seventh and twelfth lunar months. Interestingly, this effect was observed across both genders and in both urban and rural areas, highlighting the widespread influence of cultural beliefs on healthcare utilization in Taiwan. The gender-specific difference in surgical numbers during the seventh lunar month is particularly noteworthy, suggesting that certain cultural beliefs may be more ingrained in certain gender groups.

The findings in our study are consistent with other research that indicates that cultural beliefs can have a significant association on medical decision making. To address these issues, healthcare providers can take several steps. One approach is to provide patient education, which can help increase awareness of the risks and benefits of various medical procedures and medications. This can also help patients understand the importance of adhering to their medical treatments and following recommended health behaviors. Moreover, building trust and understanding with patients and their families is also essential. This can involve taking the time to listen to their concerns, respecting their beliefs and cultural practices, and providing individualized care that takes into account their unique cultural backgrounds. By doing so, healthcare providers can better serve the needs of their patients and promote positive adherence and health outcomes, even during culturally sensitive times such as the lunar ghost month or Chinese New Year.

The strength of this study is that we used a population-based and nationwide database, which provides a large and diverse sample of medical-seeking behavior in Taiwan. This allows for a more accurate reflection of the impact of cultural beliefs on medical decision making during particular Chinese lunar festivals and events. However, there are also some limitations to this study that need to be considered. Firstly, the data were obtained retrospectively. This can limit the scope of the analysis and make it difficult to draw definitive conclusions about the relationship between cultural beliefs and medical decision making during ghost months. Additionally, the data we collected lacked socioeconomic status and the level of knowledge of the participants. These factors could also play a role in medical decision making and may influence the observed results. Therefore, it is important to conduct further analysis to investigate the potential impact of these factors and to explore whether the observed reduction in surgeries during different lunar months is a true reflection of the impact of cultural beliefs. An advanced questionnaire for citizens and medical providers might be needed in the future. Further studies could also explore the potential impact of this reduction on surgical complication rates and patient outcomes.

## 5. Conclusions

The belief that the lunar ghost month is inauspicious for major surgical procedures, including cataract surgery, is held among Taiwanese people. As a result, many Taiwanese may avoid elective surgery during this month. In the twelfth lunar month, the number of cataract surgeries also decreased. It might be related to the coming of the Chinese New Year. During this period, individuals tend to delay or avoid seeking medical treatments due to traditional customs and practices that are considered necessary, as well as the desire to spend time with family.

The findings in this study emphasized the importance of cultural beliefs in medical decision making in Taiwan; therefore, it may be beneficial for the authorities to consider these factors when developing medical policies and allocating resources.

## Figures and Tables

**Table 1:** The average number of cataract surgeries (per 1,000,000 inhabitants) in each Chinese lunar month for different genders and living areas.

Chinese Lunar Month	Male	Female	*p* Value	Urban	Rural	*p* Value
1st	110.25 ± 26.51	129.60 ± 26.82	0.020 *	128.85 ± 29.38	111.00 ± 24.26	0.079
2nd	110.90 ± 16.13	135.05 ± 28.11	<0.001 *	130.45 ± 31.00	115.50 ± 16.70	0.120
3rd	109.00 ± 17.14	146.45 ± 23.54	<0.001 *	131.45 ± 31.35	124.00 ± 23.96	0.588
4th	102.75 ± 20.91	124.05 ± 30.55	<0.001 *	117.40 ± 32.50	109.40 ± 22.80	0.337
5th	96.20 ± 18.40	120.70 ± 20.09	0.001 *	110.60 ± 24.48	106.30 ± 21.20	0.588
6th	103.40 ± 17.30	123.15 ± 14.91	0.001 *	117.60 ± 19.14	108.95 ± 17.92	0.218
7th	80.15 ± 14.80	74.65 ± 12.70	0.193	84.55 ± 14.26	70.25 ± 9.24	<0.001 ^§^
8th	105.40 ± 17.41	127.05 ± 16.16	0.001 *	119.45 ± 22.83	113.00 ± 16.37	0.304
9th	109.80 ± 16.73	133.40 ± 19.14	0.001 *	125.85 ± 23.08	117.35 ± 19.21	0.239
10th	108.80 ± 14.08	141.00 ± 18.42	<0.001 *	130.35 ± 23.64	119.45 ± 21.49	0.203
11th	104.05 ± 16.96	129.65 ± 20.24	<0.001 *	122.85 ± 27.00	110.85 ± 15.41	0.172
12th	60.50 ± 21.18	67.50 ± 27.52	0.323	68.40 ± 24.94	59.60 ± 23.86	0.330
Mean	100.10 ± 11.55	121.02 ± 14.70	<0.001 *	115.65 ± 18.99	105.47 ± 12.83	0.070

* Statistically significant (*p* value < 0.05) in comparison with the number of surgeries for different genders (Mann–Whitney U test). ^§^ Statistically significant (*p* value < 0.05) in comparison with the number of surgeries in different living areas (Mann–Whitney U test).

**Table 2:** Mixed model analysis of the average number of cataract surgeries for different genders and living areas.

Living Area	Factors	F ^†^	*p* Value
Urban	1st lunar month	0.276	0.604
	7th lunar month	35.023	<0.001 *
	12th lunar month	2.265	0.146
Rural	1st lunar month	0.199	0.660
	7th lunar month	37.079	<0.001 *
	12th lunar month	3.864	0.064

* *p*-value less than 0.05 is considered the threshold for statistical significance. The average number of surgeries performed in the 2nd, 3rd, 4th, 5th, 6th, 8th, 9th, 10th, and 11th lunar months is set as the baseline to which the average numbers of surgeries in the 1st, 7th, and 12th lunar months are compared, respectively. ^†^ The F value represents the statistic for testing whether the average of numbers of surgeries are equal between compared groups.

## Data Availability

Restrictions apply to the availability of these data. Data was obtained from the National Longitudinal Health Insurance Database 2000 in Taiwan and are available from the authors with the permission of Longitudinal Health Insurance Database 2000.
